# On the need for abstract, deep reinforcement learning models in neuroscience

**DOI:** 10.3389/fninf.2026.1729805

**Published:** 2026-01-27

**Authors:** Santina Duarte, Xena Al-Hejji, Edgar Bermudez Contreras, Eric Chalmers

**Affiliations:** 1 Department of Mathematics and Computing, Mount Royal University, Calgary, AB, Canada; 2 Canadian Centre for Behavioral Neuroscience, University of Lethbridge, Lethbridge, AB, Canada

**Keywords:** abstraction, artificial neural network (ANN), deep learning, modeling, reinforcement learning

Deep Reinforcement Learning (DRL) algorithms combine artificial neural networks with reward-based learning processes, and are a useful analog of reward-based information processing and dopamine-driven learning in the brain (Botvinick et al., 2020). In our work, we have successfully used DRL algorithms to recreate features of mood disorders observed *in vivo*. However, when we present our results, we sometimes hear the objection that DRL algorithms are so much *simpler* than the brain, that they can't possibly be useful tools for studying it. For example, reviewers point out (quite correctly) that plasticity in the artificial neural network is achieved through backpropagation, which is computationally convenient but biologically implausible. Or that bias terms in the artificial network have no obvious neural correlate. Or that DRL ignores the roles of genetics, specific brain structures, signaling methods, etc.

At the risk of sounding ungrateful to our critics, (on the contrary, we would be nowhere without them!) we will here argue for the importance of abstract models like DRL in science, and show that DRL is already being used to generate testable neuroscientific hypotheses.

## Of models and metaphors

In science we understand complex phenomena through various models, which exist on a spectrum from high to low abstraction. High-level models abstract away detail in order to highlight “big picture” principles. They could include block diagrams, schematics, or mathematical or other models that describe a system's behavior but abstract away implementation details (roughly corresponding to the Algorithmic level in Marr's levels of analysis). Lower-level models include more details of the phenomena, and so obscure overarching principles - these models are closer to Marr's Implementation level of analysis. Examples may include animal models,^1^ or mathematical models of specific cellular interactions.

Every model is a kind of metaphor that helps us understand *part* of a complex phenomenon. To ask whether a metaphor (or a model) is “correct” is non-sensical; to quote the aphorism attributed to George Box, “All models are wrong, but some are useful” (Box, 1976).^2^ Consider a schematic diagram of brain structure interactions (high abstraction), and an animal model of a human disorder (low abstraction)—neither are completely accurate representations, but each is useful in the right situation: imagine trying to teach neuroscience without the diagram, or test drugs without animal models, or teach neuroscience with *only* animal models!

Pursuing more detailed/accurate models for their own sake can distract us from more fundamental concerns. Jorge Luis Borges warned against this in a 1946 short story titled *On Exactitude in Science*. It describes a fictional empire whose preoccupation with perfection in cartography led them to create ever larger and more detailed maps. Their consummate creation was a map as large as the empire itself, and which corresponded point-for-point with it. Obviously, this map was worse than useless. It was abandoned by the next generation, and the story concludes tragically: “in all the land there is no other relic of the disciplines of geography” (Borges, 1999).

It's possible that the phenomena we care about most (consciousness, desire, love, etc) cannot be found in the lowest-level mechanisms of information processing. Rather, they may themselves be higher-order phenomena that are best understood with the help of more abstract models. If so, a preference for more detailed models—to the exclusion of higher-level metaphors—seems doomed to end in the frustration hinted at by Borges' story. Yet, and as was presumably the case in Borge's day, there are a few life scientists on this trajectory now. To quote Douglas Hofstadter's lament: “how widespread is the tacit assumption that the level of the most primordial physical components of a brain must *also* be the level at which the brain's most complex and elusive mental properties reside” (Hofstadter, 2008). Science requires thought across the full spectrum of abstraction. Very occasionally it may even be possible to consider multiple levels at the same time—ecent work by Shine et al. illustrates how computational models may facilitate this (Shine et al., 2021).

DRL algorithms exist at an intermediate level of abstraction, much lower than block diagrams but much higher than animal models. This gives them the unique ability to illustrate core features of learning, while glossing over specifics of their neural implementation. The latter point sometimes dissuades scientists who are accustomed to working at a level closer to implementation. But most neuroscientists, who are comfortable working at higher levels of abstraction, see that it is *because* DRL models fly above the level of implementation details that they offer a unique computational perspective on intelligent behavior. This perspective has allowed these scientists to generate and test a variety of meaningful hypotheses about biological learning. We conclude with a few examples.

## Successes of deep reinforcement learning in neuroscientific discovery

Song et al. (2017) built a DRL model that reproduced observations from an early study conducted with monkeys (Padoa-Schioppa and Assad, 2006). That earlier study had found particular neurons in the orbitofrontal cortex that seemed to encode the inferred economic value of the monkey's preferred choices in several tasks. Song et al. found that reward-based training of artificial neural networks caused certain artificial neurons to take on similar roles. Their computational model predicted a role for value representations in the brain which support learning of a task, but exist independently of its execution (it would have been difficult to reach this hypothesis through *in vivo* experiments).

Banino et al. (2018) discovered that when an artificial neural network with recurrent connections was trained to do path integration, neurons in the network began to fire at regular spatial intervals—resembling the behavior of entorhinal grid cells. They next found that a DRL model based on this network could effectively perform vector-based navigation, even in unfamiliar or dynamic environments. These findings support neuroscientific hypotheses that grid cells enable vector-based navigation.

In their work with DRL, Wang et al. (2018) noticed an interplay between relatively slow RL processes and faster recurrent network dynamics, which allows the system to learn general characteristics of a family of tasks, and then quickly solve new tasks from that same family—similar to how humans can transfer knowledge gained in one task to other, similar tasks. They used these results to explain neuroscientific findings that challenged traditional notions of dopamine-driven RL, such as the observation that the prefrontal cortex and dopamine neurons both seem to encode reward-based signals that classical RL models ascribe solely to dopamine. Follow-up work by Jensen et al. (2024) augmented Wang's DRL system with the ability to sample imagined action sequences based on previous experience. Jensen's model provides a new computational account of planning and hippocampal replay in mammals.

Wijmans et al. (2023) discovered that if a DRL agent's neural network was endowed with recurrent connections, this recurrence allowed map-like representations and collision-detection neurons to emerge in the network. These effects were observed even when the agents were blind—with no sensory input other than egomotion—predicting that network recurrence is a sufficient condition for the emergence of “mental maps.” These findings have interesting implications for understanding the mechanistic basis of navigation in mammals.

While the above studies observed neural phenomena in DRL models, a study by Dabney et al. (2020) is an interesting example of the reverse. Classical RL models use a reward-prediction-error signal [thought to be conveyed in the brain by phasic dopamine activity Schultz et al., 1997; Montague et al., 1996] to drive learning. While this signal is typically a scalar—representing a single, point prediction of expected value—artificial intelligence practitioners have long realized the value of using a probability distribution over *possible* values instead. This allows the agent to reason over possible futures, and better manage risk. Inspired by this idea, Dabney et al. searched for and found similar distributional codes in mouse brains.

Finally, our own work has used DRL to create computational models of major depressive disorder (Chalmers et al., 2024) and schizophrenia (Al-Hejji et al., 2025). After impairing a DRL agent's neural network in ways analogous to what is observed in these disorders, we observed similar behaviors to the ones exhibited by depressed and schizophrenic humans and animals. In both cases, deep examination of the DRL model suggested ways to reconcile competing ideas about the disorders' pathologies (e.g., the model suggests a way to reconcile dopaminergic and neurodevelopmental theories of schizophrenia) and offered useful insight to guide treatment research. These results were possible partly because making alterations in a DRL model is so much easier than attempting the same alterations *in vivo*.

## In conclusion

Advances in AI have largely been driven by engineering goals and applications, which has led to the perception that AI approaches, such as DRL, offer little value in understanding biological systems. However, as the examples mentioned above (and many more) show, this view is increasingly outdated. When used appropriately, DRL and related approaches provide a useful framework to study the dynamic interplay between agents and their environments, shedding light on fundamental principles of how representations, learning, memory, and decision making work in biological systems. Such frameworks offer a way to generate and formalize hypotheses, test mechanisms, and generate insights that are difficult to obtain from experimental work alone. We hope that this perspective gains broader recognition, allowing deep reinforcement learning and related methods to enrich neuroscience research.

## Footnotes

1 Though even animal models are too abstract to elucidate some human disorders. “High” and “low” abstraction are relative, and a model that is not abstract enough for one purpose could be too abstract for another.

2 This line is often attributed to Box. A more faithful (and pertinent) quote from his 1976 paper is: “Since all models are wrong the scientist cannot obtain a ‘correct' one by excessive elaboration… Just as the ability to devise simple but evocative models is the signature of the great scientist so overelaboration and overparameterization is often the mark of mediocrity”.
